# Human cytomegalovirus is present in the odontogenic epithelium of ameloblastoma

**DOI:** 10.1080/20002297.2021.1874699

**Published:** 2021-01-19

**Authors:** Mohammed Amjed Alsaegh, Sudhir Rama Varma, Alaa Muayad Altaie, Shengrong Zhu

**Affiliations:** a Department of Oral and Craniofacial Health Sciences, College of Dental Medicine, University of Sharjah, Sharjah, UAE; b Department of Oral and Maxillofacial Surgery, Tongji Hospital of Tongji Medical College, Huazhong University of Science and Technology, Wuhan, P.R. China; c Department of Clinical Sciences, College of Dentistry, Ajman University, Ajman, UAE; d Sharjah Medical Research Institute, Medical College, Sharjah University, Sharjah, UAE

**Keywords:** HCMV, cytomegalovirus, odontogenic tumor, ameloblastoma, dentigerous cyst, keratocyst, odontogenic

## Abstract

**Background:** The factor behind the activation of the remnant odontogenic tissues and development of odontogenic cysts and tumors is poorly understood.This study aimed to investigate the presence of human cytomegalovirus (HCMV) in dentigerous cyst (DC), odontogenic keratocyst (OKC), and ameloblastoma (AB).

**Methods:** The study included 41 samples, which distributed into DC (n=13), OKC (n=12), and AB (n=16). Conventional PCR assay and IHC analysis were used to detect the HCMV-DNA and HCMV glycoprotein B (HCMV-gB) respectively.

**Results:** HCMV-DNA was detected in 10 samples (62.5%) of AB, four samples (30.8%) of DC, and three samples (25 %) of OKC respectively (χ^2^ test = 1.195, p= 0.247). Meanwhile, HCMV-gB was found in 12 (75%) of AB, in 2 (15.4%) of DC, and absent in OKC (0.0%) (χ^2^ test = 4.122, p= 0.042).

**Conclusions:** The high prevalence of HCMV inside the odontogenic epithelium of AB could indicate a possible role of the virus in the oncogenesis and/or oncomodulation of the AB. Additionally, we recommend the IHC for the detection of HCMV in the odontogenic tumors like AB.

## Introduction

Odontogenic lesions are a group of diseases that originate from the remnant odontogenic tissues in the jaws and cause morbidity to the maxilla and the mandible. While these tissues are present in all individuals, the factor behind their activation and further development of odontogenic cysts and tumours is poorly understood.

HCMV is the most frequent human pathogen in the family of the herpes viruses. It infects 50–100% of the general adult population. Molecularly, HCMV is a double-stranded DNA virus enclosing 165 genes. These genes encode viral proteins which are responsible to the virus latency and virulence. The HCMV proteins are mimic and interact with the human cellular proteins[1]. HCMV has a high cellular tropism, it infects different host cells like monocytes/macrophages, polymorphonuclear leukocytes, T lymphocytes, fibroblasts, epithelial, and endothelial cells[2]. The virus replicates inside the nucleus of the host cell and illustrates as enormous intranuclear but tiny cytoplasmic inclusion bodies in the histopathological sections. Although HCMV is known by its cytopathic effect known as owl’s eye nucleus on H&E staining, often these inclusions are not visible, and in situ hybridization and immunohistochemical (IHC) stains are used for definitive viral diagnosis[3].

Previous studies detected HCMV in different normal and pathological tissues. HCMV was detected in the periapical cyst, keratocyst[4], and in apical periodontitis [5–9]. Furthermore, HCMV was present in different tumour cells[10]. A significant relation between HCMV and tumour has been found by different independent groups of researchers. In fact, HCMV promotes tumorigenesis, exhibits immunity evasiveness, causes immunosuppressive effects in the tumour environment, and involved in anti-apoptotic and hijacks proangiogenic mechanisms[11].

Several studies used PCR for the detection of HCMV in the pathological tissues [4,6–8]. The using of PCR alone in the detection of a virus, like HCMV, who has a wide cellular tropism is lacking the information about the spatial cell distribution of the virus in the infected tissues. Accordingly, IHC approach in addition to the PCR assay were used for the investigation of HCMV in the current study. The present study aimed to investigate the presence of HCMV in dentigerous cyst (DC), odontogenic keratocyst (OKC), and ameloblastoma (AB).

## Materials and methods

This retrospective study included 41 samples. The samples were retrieved from the pathology department of Tongji Hospital, Wuhan, China. The formalin-fixed and paraffin-embedded (FFPE) samples involved 13 DCs, 12 OKCs, and 16 follicular ABs. The pathology reports and available original H&E slides of all the cases were reviewed and confirmed. All participants did not take antiviral or immunosuppressive therapies and were free of acquired immune deficiency syndrome (AIDS). The OKC samples are of the parakeratinized type, solitary, and none of them was associated with nevoid basal-cell carcinoma syndrome. The Institutional Review Board of Tongji Medical College approved this study which followed the protocol of the World Medical Association Declaration of Helsinki.

The immunostaining method was the standard streptavidin‑biotin peroxidase complex (Wuhan Boster Biological Technology, Ltd.). FFPE samples were cut into 5‑μm sections, dewaxed, rehydrated and their endogenous peroxidase activities were quenched by 3% hydrogen peroxide solution. The antigens of the tissue were unmasked by exposing the slides in heated 0.01 M citrate buffer till the boiling point in a microwave. After that, goat serum (Wuhan Boster Biological Technology, Ltd.) was used to treat the samples at room temperature for 50 min before incubating them in 1:100 diluted primary mouse monoclonal antibody that directed to the HCMV-gB at 4°C overnight (bsm-2271 M; Clone: 1F11, Beijing Bioss Co, China). Then after, the slides were incubated with 10 μg/ml of the biotinylated goat anti-mouse secondary antibody (BA1001 Wuhan Biological Technology, Ltd.) for 2 h at room temperature. This step was followed by staining with 20 μg/ml streptavidin‑biotin‑peroxidase complex. Consequently, 3,3ʹ‑diaminobenzidine substrate was used to develop the sections which are then counter-stained with Mayer’s hematoxylin. The negative controls were passed through all the previous steps, but phosphate‑buffered saline was used instead of the primary antibody.

Each slide was investigated totally, the positive result was identified as a yellowish-brown precipitate in the nucleus and cytoplasm of the odontogenic epithelium using high power field. Meanwhile, absence or questionable staining in the odontogenic epithelium were deemed as negative results.

Each FFPE sample was cut into five pieces of 10 μm thick sections for the conventional PCR analysis. These pieces were deparaffinized, rehydrated and the DN32 DNA extraction kit (Aidlab. Co. LTD, Beijing, China) was used to extract the DNA from them. The suitability of the extracted DNA for further molecular analysis was assessed using primers that recognize the albumin gene. The final PCR products was 159 bp in size using the primers for HCMV detection that adopted from Andric et al [4]. as follows: HCMV F: 5ʹ-CCACCCGTGGTGCCAGCTCC-3ʹ and HCMV R: 5ʹCCCGCTCCTCCTGAGCACCC-3ʹ.

The PCR reaction was performed in a final reaction volume of 25 μL containing 3 μL of the isolated DNA solution, 15 pmol of forward and reversed primers, 200 mmol/L of each deoxynucleoside triphosphate, and 12.5 μL of 2 × Taq MasterMix (Aidlab Co. LTD, Beijing, China). The DNA Initial denaturation was performed at 94°C for 3 min, followed by 30 cycles of 30 s for each of denaturation at 94°C; annealing at 56°C; and elongation at 72°C, while the final extension was achieved at 72°C for 5 min. Each PCR experiment was performed with positive and negative controls. Lymphoid cell lines containing HCMV were used as positive controls, whilst a PCR mixture containing 3 μL distilled water instead of sample was used as a negative control. The final 159-bp products were visualized using ethidium bromide staining running through 3% agarose gel.

Data were analyzed using the Statistical Package for the Social Sciences (SPSS) 19.0 software (IBM SPSS, Armonk, NY, USA). The differences were detected using chi-square test, while Kappa statistical test was used to assess the degree of agreement between IHC and PCR results. P value of <0.05 was deemed significant.

## Results

The study involved 41 cases. Among them, 18 cases were females (43.9%) and 23 cases were males (56.1%). The mean age of patients was 37.24 years. The most common site of involvement was the mandible (26 cases; 63.4%), whereas the maxilla was involved in 15 cases (36.6%). The relevant clinical features of the participants in the current study are summarized in Table 1.

**Table 1.** Clinical features of the studied cases

Variables	Total	Dentigerous cyst	Odontogenic keratocyst	Ameloblastoma
Patients (n)	41	13	12	16
Age (years) Mean Range	37.24 12–74	40 12–74	39.42 15–66	33.38 12–70
Gender, n (%) Male Female	23 (56.1%) 18 (43.9%)	10 (76.9%) 3 (23.1%)	5 (41.7%) 7 (58.3%)	8 (50%) 8 (50%)
Location, n (%) Mandible Maxilla	26 (63.4%) 15 (36.6%)	3 (23.1%) 10 (76.9%)	8 (66.7%) 4 (33.3%)	15 (93.8%) 1 (6.3%)

HCMV-DNA was detected in four samples (30.8%) of DCs, three (25%) of OKCs, and 10 (62.5%) of ABs consequently (Table 2). Statistical analysis using chi-square test showed a non-significant difference in the detection of HCMV-DNA among the studied samples (χ2 test = 1.195, p = 0.247).

**Table 2.** The presence of HCMV-DNA and the expression of HCMV-gB in the studied groups

Samples	HCMV-DNA	HCMV-gB
Dentigerous cyst (*n*= 13)	+ve (*n*= 4) 30.8% -ve (*n*= 9) 69.2%	+ve (*n*= 2) 15.4% -ve (*n*= 11) 84.6%
Odontogenic Keratocyst (*n*= 12)	+ve (*n*= 3) 25% -ve (*n*= 9) 75%	+ve (*n*= 0) 0.0% -ve (*n*= 12) 100%
Ameloblastoma (*n*= 16)	+ve (*n*= 10) 62.5% -ve (*n*= 6) 37.5%	+ve (*n*= 12) 75% -ve (*n*= 4) 25%

**Figure 1.**
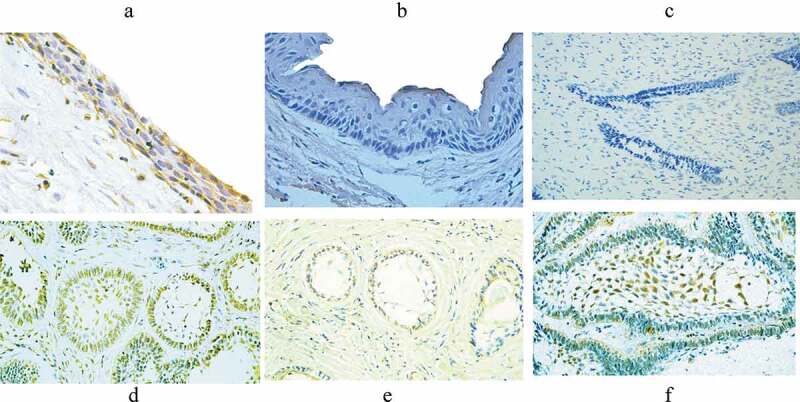
Immunohistochemical expression of HCMV-gB in the studied odontogenic lesions. The positively stained cells in dentigerous cyst were distributed throughout the epithelial wall (A), absence of staining in odontogenic keratocyst (B), negative staining in ameloblastoma (AB) (C), heavy expression of HCMV-gB in both the peripheral columnar cells and the stellate reticulum‒like cells of AB (D), more positive expression in the peripheral columnar cells of AB (E), and (F) more positive expression in the stellate reticulum‒like cells of AB

HCMV-gB was immunohistochemically identified as a yellowish to brown precipitate that located mainly in the nucleus and less frequently in the cytoplasm of the studied odontogenic epithelial cells. The location of HCMV-gB expression was in both the peripheral columnar cells and stellate reticulum‒like cells of Abs, while positively stained cells in DC were located throughout the layers of the epithelial wall (Figure 1).

A positive HCMV-gB protein expression was found in 12 cases (75%) of AB. In DC samples, only two cases (15.4%) were showed positive HCMV-gB staining. Meanwhile, all OKC samples showed a negative staining of HCMV-gB (Table 2). Chi-square test showed significantly higher expression of HCMV-gB in AB than in DC and OKC (χ2 test = 4.122, p = 0.042). Kappa coefficient test showed moderate agreements (κ = 0.433, p = 0.005) between PCR and IHC results in the total-studied samples.

## Discussion

The factor behind the activation of the remnant odontogenic epithelium in the jaws and further development of odontogenic cysts and tumours is poorly understood. This article investigated the presence of HCMV in the odontogenic cysts and tumours. To the extent of our knowledge, this is the first report dealing with the presence of HCMV in AB and DC. Nevertheless, there is only one previous article that investigated the presence of HCMV in OKC[4].

Both viral DNA and protein was investigated in the current study. Accordingly, HCMV-DNA was detected as a non-significantly different in AB, DC, and OKC. The prevalence of HCMV-DNA in our odontogenic lesion samples is comparable to other studies that conducted in the odontogenic cysts and periapical granulomas [4,7]. However, a more frequent detection was observed when the odontogenic cyst has a history of previous episodes of acute infection [4] and in symptomatic and large periapical granuloma [6,7]. Despite its comparable PCR results, the current study showed a significant difference in the HCMV-gB expression in the odontogenic epithelium of the different groups of the studied lesions. This could be attributed to the fact that detection of HCMV-DNA is reflecting the total cellular presence of the virus in the studied lesions rather than its real infection of the odontogenic epithelium of the studied cysts and tumours. In fact, HCMV is the most common herpes virus that causes infection for human where it infects 50–100% of the general adult population[1]. Inside host, HCMV infects different immune cells like monocytes/macrophages, polymorphonuclear leukocytes, and T lymphocytes[2]. Consequently, the HCMV infected leukocytes could invade the odontogenic cysts and tumours leading to the on par PCR recognition of HCMV-DNA in these studied odontogenic lesions. In accordance to this opinion, Sabiti et al. found that HCMV has infected mainly the inflammatory cells in dental periapical lesions, where 40% of the virus was detected in the monocyte/macrophage and 54% in the T lymphocytes using multicolour flow cytometry[12]. Leukocytes are heavily infiltrating the periapical granuloma. This could be the reason behind the detection of HCMV-DNA in the periapical lesions of the previous studies using PCR assay [6,7].

The current study recommended that a possible role of HCMV in odontogenic lesions should rely on the IHC results rather than the PCR one. The spatial distribution of HCMV and effect inside the odontogenic epithelium is better identified using IHC. Therefore, IHC detection of HCMV is more specific method than non-spatial detection of HCMV-DNA using PCR.

Our results showed that PCR assay is more sensitive in detecting HCMV than IHC in both DC and OKC. Alternatively, IHC was more sensitive in AB samples (positivity was 75% in IHC and 63% in PCR). AB is an odontogenic tumour while DC and OKC are not. It was found that PCR and sequencing methods of HCMV detection inside tumour has a generally unresolved technical problems to detect the HCMV-DNA in specimens of tumor[11]. These problems were attributed to the high genetic discrepancy of HCMV genome and the inherited difficulties associated with Taq polymerases to read the DNA of the HCMV in the tumour tissues[13]. Furthermore, some negative expression of HCMV-DNA in different tumours could be due to that only part of the HCMV genome, which is not targeted by PCR methodology, is present in the tumour tissues[10].

HCMV replicates inside the nucleus of the host cells and illustrates as enormous intranuclear and tiny cytoplasmic inclusion bodies. In histopathological sections, HCMV is identified by viral cytopathic effect known as owl’s eye nucleus on H&E staining. However, these inclusions are usually not visible, and in situ hybridization and IHC stains are used for the definitive diagnosis[3]. In the present study, the IHC detection was for HCMV-gB. HCMV-gB is an ample protein, which located in the envelope of the virus. It is regarded as a strong immunogenic protein and it is essential for the infectivity of the virus. Specifically, HCMV-gB provides the virus with the cell fusion activity that required for infection of all cell types. Meanwhile, other envelope proteins are involved in infectivity of only specific cell types[14]. Although the location of the HCMV-gB expression was mainly nuclear, the cytoplasmic expression was also identified in the current study. Indeed, the cytoplasmic expression of HCMV proteins are often present in the infected tumour cells. This type of expression is rarely observed in other infected tissues, suggesting an alternative behaviour of HCMV in tumour cells or even the existence of unique strains of HCMV that associated with the tumour [11]. Peculiarly, this cytoplasmic expression of HCMV was also found in samples of apical periodontitis[2].

Several studies detected HCMV in different pathological lesions of the jaws. A previous study revealed a significant presence of HCMV in samples of periapical cyst and OKC[4]. Besides, HCMV was detected in the apical periodontitis using both PCR [6,7] and IHC [2,8]. Interestingly, HCMV has a higher prevalence in the symptomatic and/or the large chronic periapical lesions than the asymptomatic and/or small lesions [6,7]. Furthermore, HCMV was present in the periapical lesion of teeth with an intact crown, indicating that the infection does not originate from the mouth, but rather transferred to this location by immune cells[5]. Contrary, other previous studies found that HCMV infection was rare in apical lesions [15] and absent in the periapical abscess[16].

The IHC results of our study did not reveal the presence of HCMV in the odontogenic epithelium of OKCs. This result disagrees with a previous study that found the presence of HCMV in 60% of OKC samples[4]. Possibly, this disagreement is related to the methodology used in the detection of HCMV. Andric et al. used the PCR method to detect the HCMV-DNA, while we recommended the IHC as an actual spatial detection of HCMV. Noteworthy, we also detected HCMV-DNA in 25% of OKC samples using the same primer recommended by Andric et al [4]. The PCR positivity could be attributed to the virus presence in the connective tissue capsule of the studied OKCs. In DC samples, we found a scarce expression of HCMV-gB in just two cases (15%). However, the virus was detected in 30% of the studied DC samples using PCR assay. The light and rare expression of HCMV-gB in DC recommends further spatial studies of the HCMV expression in this type of the odontogenic cysts, considering the lack of reports dealing with HCMV investigation in DC.

A higher expression of HCMV viral protein in AB than in DC and OKC was found in the present study. One could speculate a possible role of HCMV in the etiopathogenesis and/or oncomodulation of the AB. AB is a true tumour, while DC is an odontogenic cyst and OKC was reclassified as an odontogenic cyst in the last WHO classification in the year 2017 from what it was known as a tumour. More investigations are needed to elaborate the possible role of the virus in the oncogenesis or oncomodulation of AB. The high prevalence of HCMV-gB inside the odontogenic epithelium of the AB samples could indicate the presence of an active virus involvement rather than just concomitant presence of HCMV-DNA in cells other than odontogenic epithelium of the studied samples. Evidence indicated a role of HCMV in both oncomodulation and oncogenesis of the tumour [10]. Actually, HCMV promotes the survival of its host cells through encoding different viral proteins that hindering apoptosis and securing sufficient viral replication[17]. Hence, we cannot exclude a possible role of HCMV that helps the hosting odontogenic epithelial cells of AB to survive and escape the apoptosis. Notwithstanding, a just incidental presence of HCMV inside the odontogenic epithelium of the odontogenic lesions is still a prospect that also needs to further investigations.

It is well-known that HCMV pathology is linked to its broad cell tropism. Thereby, it infects most human tissues and organs. HCMV can be found in more than 90% of total human epithelial tumours. It has been speculated that positive PCR results of HCMV-DNA could reflect an opportunistic infection of HCMV in the frequently immunosuppressed cancerous patients[10]. Furthermore, detection of the viral DNA could reflect a contamination that happened during the surgical procedure which is the main current treatment for DC, OKC, and AB. However, the IHC detection of the HCMV-gB inside the tumor cells, like in the current study, could give more clues for a possible role of HCMV in the tumor initiation and progression.

The consequence of HCMV infection differs according to the kind of the infected cells. Comparable to odontogenic epithelium that studied in our report, epithelial cells provide less degree of viral replication as the virus reveals a chronic infection and makes little but persistent viral shedding. This is not the case in other cell types like fibroblasts and smooth muscle cells where the HCMV replication is in large quantities leading to almost heavy viral progeny[18]. The chronic pattern of HCMV infection to the epithelial cells simulates the slow-growing feature of AB.

The viral gB part has a pivotal role for the HCMV infection of all cell types. Hence, McVoy et al. suggested that gB as an interesting structure for the development of a drug that targeting HCMV. Additionally, gB is qualitatively more conserved than the rest of the HCMV genome[14]. This suggestion gives a clinical significance for our study that targeted and found high expression of HCMV-gB in ABs. Traditionally, the clinical management of HCMV is mostly focused on tissue transplant cases. The presence and possible relation of HCMV in an odontogenic tumour could further investigate the strategy of HCMV therapy in the tumour cases.

A further study is indicated to explore the molecular effects and pathways of HCMV in the odontogenic lesions. This will help in better understanding of the virus role in AB and excludes the incidental and passive presence of HCMV in the related lesion.

## Conclusion

Although the incidental presence of HCMV cannot be excluded, the high prevalence of HCMV inside the odontogenic epithelium of AB could indicate a possible role of the virus in the oncogenesis and/or oncomodulation of the AB. Additionally, we recommend the IHC for the detection of HCMV in the odontogenic tumours like AB.
